# Supplementary material for: Boundary conductance in macroscopic bismuth crystals
Source: Nat Commun. 2022 Jan 11;13:189. doi: 10.1038/s41467-021-27721-7 (PMC8752747; doi:10.1038/s41467-021-27721-7)
Supplement: Supplementary file 1 — Supplementary information [file 41467_2021_27721_MOESM1_ESM.pdf]

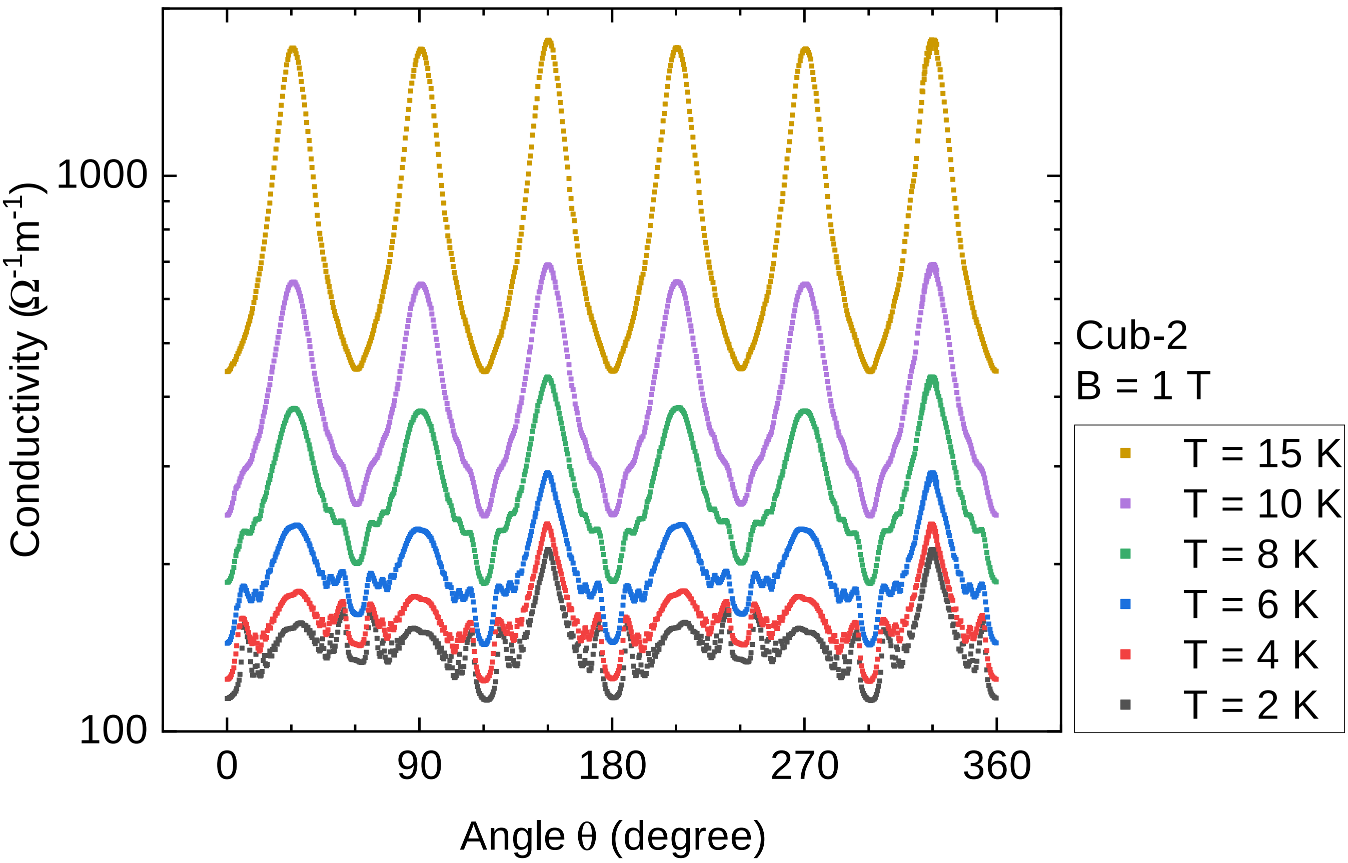

Supplementary Figure 1: Angle-dependent magnetoconductivity at B = 1 T and several temperatures in sample Bi-Cub-2. Below 8 K, quantum oscillations and shape effect (excess conductivity at specific angles of $\Theta = 60$, 150, 240 and 330 degrees) are clearly visible. Upon warming, both disappear concomitantly.

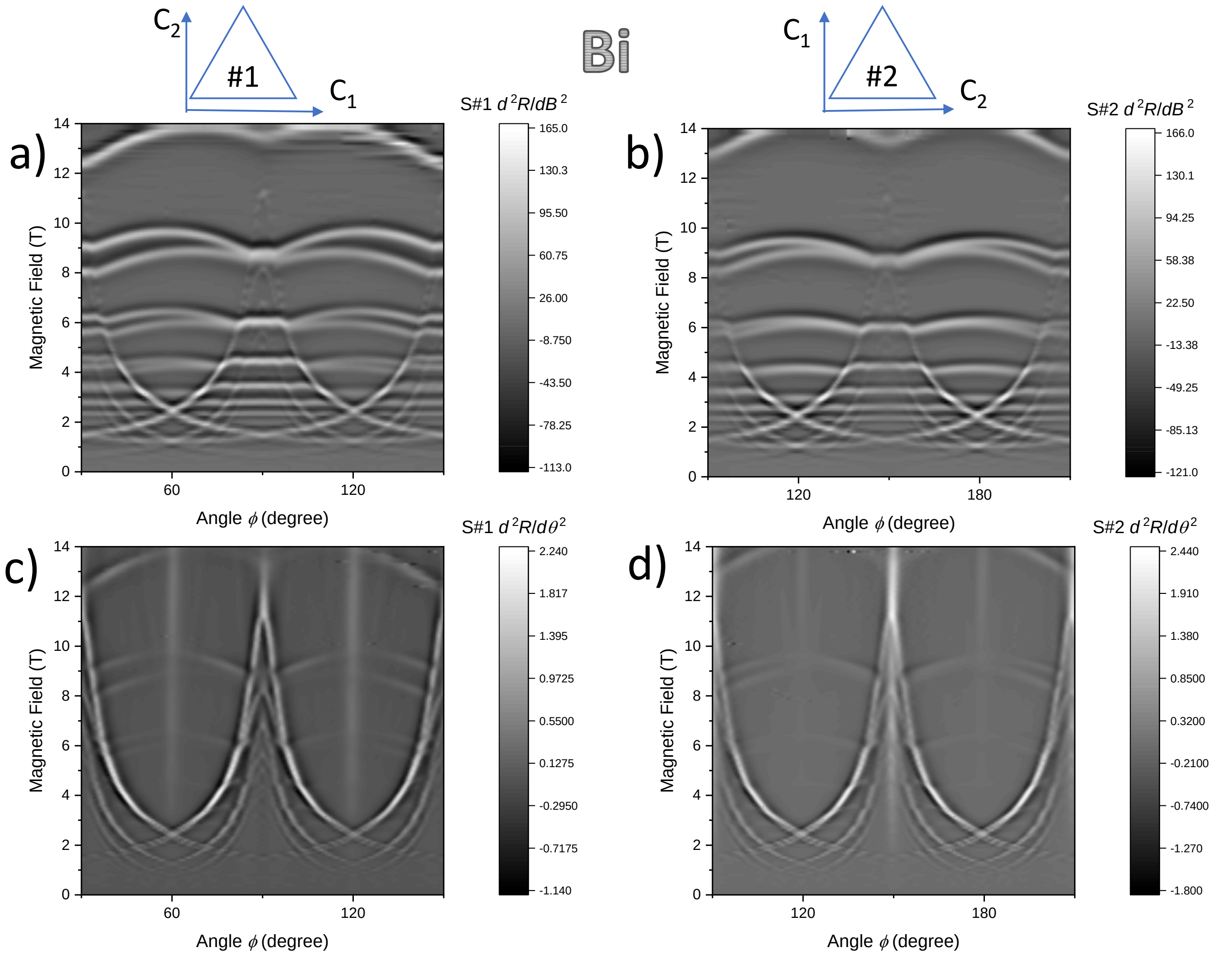

Supplementary Figure 2: The second derivative of the magnetoresistance with respect to magnetic field in sample 1 (a) and in sample 2 (b) and the second derivative of magnetoresistance with respect to angle in sample 1 (c) and in sample 2 (d). The quasi-horizontal lines are caused by the evacuation of the hole-like Landau levels. Those with a sharp angle dependence correspond to electron-like Landau levels. The Landau spectrum is entirely identical in the two samples.

I. SUPPLEMENTARY NOTE 1: RESIDUAL RESISTIVITY

In this section, we compare the residual resistivity of the Bi samples studied in this work with those reported in previous studies. As seen in table 1 in the main text, the residual resistivity of the present samples range from 0.18 to 0.4 $\mu\Omega\,$cm. Note that this corresponds to the longitudinal resistivity along the trigonal C_3 axis. For the cleanest sample, we found $\rho_{33}(2\text{ K}) = 0.18\ \mu\Omega\,$cm.

The first study of the low-field galvanometric tensor in Bi was performed on a crystal with $\rho_{33}(4.2\text{ K}) = 0.8\ \mu\Omega\,$cm and $\rho_{33}(4.2\text{ K}) = 0.74\ \mu\Omega\,$cm [1]. This work was followed by Hartman's more extensive study on a crystal with $\rho_{33}(4.2\text{ K}) = 0.321\ \mu\Omega\,$cm and $\rho_{33}(4.2\text{ K}) = 0.291\ \mu\Omega\,$cm [2].

Soviet authors often specified the residual resistivity ratio; RRR $= \rho(300\text{ K})/\rho(4.2\text{ K})$. The highest reported RRR range from 625 [3] to 650-680 [4], to be compared with what we find in our best sample (RRR $= \rho(300\text{ K})/\rho(2\text{K}) =$ 683).

In contrast, more recent studies of magnetoresistance in high magnetic field [5, 6] were performed on small crystals cut from bigger pieces. These small crystals had a RRR of $\simeq 150$ and a residual resistivity of $\rho(2\text{ K}) \simeq 1\ \mu\Omega\,$cm. Unsurprisingly, their magnetoresistance was lower than the magnetoresistance of the samples studied here.

II. SUPPLEMENTARY NOTE 2: ONSET OF THE SHAPE EFFECT

Figure 1 shows the angle-dependent magnetoconductivity of sample Bi-Cub-2 at a field of 1 T and several temperatures. The excess conductivity for B || surface (see Fig. 4d in the main text for the geometry of the sample) is absent at 15 K and appears in a smooth way upon cooling. The same is true for quantum oscillations. Below 15 K, they become visible at first close to $\theta = n\pi/3$, finally showing a very complex behaviour at T = 2 K.

The fact that quantum oscillations and the shape effect appear at the same temperature and seem to evolve in a similar way upon cooling strongly suggests that they require the same condition to occur. Therefore, one can safely conclude that the $\omega_c\tau_D \approx 1$ criterion has to be fulfilled for the excess conductivity to be present. Moreover, this supports the conclusion that the shape effect is a quantum effect and not a semi-classical one.

III. SUPPLEMENTARY NOTE 3: LANDAU SPECTRUM

The angle-dependent Landau spectrum remains identical in the two triangular prisms with faces tailored along different crystallographic planes. This is shown in Fig. 2. By taking the second derivative of the magnetoresistance and displaying the variation of its amplitude in the (field, angle) plane one can see bright lines corresponding to the angle-dependent Landau spectrum of bismuth [7, 8]. Taking the second derivative respective to the field reveals quasi-horizontal lines corresponding to the hole Landau levels, while taking the second derivative respective to the angle reveals mostly vertical lines, which correspond to the electron Landau levels.

These lines match what was previously found in studies of angle-dependent Nernst [8] and magnetoresistance [9] studies. The agreement between these experiments and the angle-dependent magnetostriction data [10] and the theoretical Landau spectrum [6–8, 11] is excellent. Therefore, we can safely assume that the difference between the two samples of different shape is not accompanied by any difference in their Landau spectrum. In spite of its complexity, this spectrum can be described in a standard treatment of electrons (with their Dirac dispersion) and holes (with their parabolic dispersion).

---

[1] R. N. Zitter, Phys. Rev. **127**, 1471 (1962), URL `https://link.aps.org/doi/10.1103/PhysRev.127.1471`.

[2] R. Hartman, Phys. Rev. **181**, 1070 (1969), URL `https://link.aps.org/doi/10.1103/PhysRev.181.1070`.

[3] V. N. Galev, V. A. Kozlov, N. V. Kolomoets, S. Y. Skipidarov, and N. A. Tsvetkova, Soviet Journal of Experimental and Theoretical Physics Letters **33**, 106 (1981).

[4] V. N. Kopylov and L. P. Mezhov-Deglin, Soviet Journal of Experimental and Theoretical Physics **38**, 357 (1974).

[5] B. Fauqué, B. Vignolle, C. Proust, J.-P. Issi, and K. Behnia, New Journal of Physics **11**, 113012 (2009), URL `https://doi.org/10.1088%2F1367-2630%2F11%2F11%2F113012`.

[6] Z. Zhu, J. Wang, H. Zuo, B. Fauqué, R. D. McDonald, Y. Fuseya, and K. Behnia, Nature Communications **8**, 15297 (2017), ISSN 2041-1723, URL `https://doi.org/10.1038/ncomms15297`.

[7] Z. Zhu, B. Fauqué, Y. Fuseya, and K. Behnia, Phys. Rev. B **84**, 115137 (2011), URL `https://link.aps.org/doi/10.1103/PhysRevB.84.115137`.

[8] Z. Zhu, B. Fauqué, L. Malone, A. B. Antunes, Y. Fuseya, and K. Behnia, Proceedings of the National Academy of Sciences of the United States of America **109**, 14813 (2012), ISSN 0027-8424.

[9] A. Collaudin, B. Fauqué, Y. Fuseya, W. Kang, and K. Behnia, Physical Review X **5**, 021022 (2015).

[10] R. Küchler, L. Steinke, R. Daou, M. Brando, K. Behnia, and F. Steglich, Nature Materials **13**, 461 (2014), ISSN 1476-4660, URL `https://doi.org/10.1038/nmat3909`.

[11] J. Alicea and L. Balents, Phys. Rev. B **79**, 241101 (2009), URL `https://link.aps.org/doi/10.1103/PhysRevB.79.241101`.
